# Impaired anti-inflammatory action of glucocorticoid in neutrophil from patients with steroid-resistant asthma

**DOI:** 10.1186/s12931-016-0462-0

**Published:** 2016-11-16

**Authors:** Meijia Wang, Pengfei Gao, Xiaojie Wu, Yuetao Chen, Yikuan Feng, Qun Yang, Yongjian Xu, Jianping Zhao, Jungang Xie

**Affiliations:** 1 Department of Respiratory and Critical Care Medicine, National Clinical Research Center of Respiratory Disease, Tongji Hospital, Tongji Medical College, Huazhong University of Science and Technology, Wuhan, China; 2 Department of Respiratory, Wuhan No.1 Hospital, Tongji Medical College, Huazhong University of Science and Technology, Wuhan, China; 3 Department Critical Care Medicine, Tongji Hospital, Tongji Medical College, Huazhong University of Science and Technology, Wuhan, China; 4 Department of Respiratory and Critical Care Medicine, National Clinical Research Center of Respiratory Disease, Tongji Hospital, Tongji Medical College, Huazhong University of Science and Technology, Wuhan, 430030 China

**Keywords:** Asthma, Steroid resistant, Neutrophil, Corticosteroids

## Abstract

**Background:**

Steroid resistant (SR) asthma is characterized by persistent airway inflammation that fails to resolve despite treatment with high doses of corticosteroids. Furthermore, SR patient airways show increased numbers neutrophils, which are less responsive to glucocorticoid. The present study seeks to determine whether dexamethasone (DEX) has different effect on neutrophils from steroid sensitive (SS) asthmatics compared to SR asthmatics.

**Methods:**

Adults with asthma (*n* = 38) were classified as SR or SS based on changes in lung FEV1% following a one-month inhaled corticosteroid (ICS) treatment. Blood samples were collected from all patients during their first visit of the study. Neutrophils isolated from the blood were cultured with dexamethasone and/or atopic asthmatic serum for 18 h. The mRNA expression of mitogen-activated protein kinase phosphatase-1 (MKP-1), a glucocorticoid transactivation target, and glucocorticoid-induced transcript 1 (GLCCI1), an early marker of glucocorticoid-induced apoptosis whose expression was associated with the response to inhaled glucocorticoids in asthma , was determined by real-time PCR, and ELISA was used to assess the pro-inflammatory cytokine IL-8 levels in the supernatant. Constitutive neutrophil apoptosis was detected by flow cytometry.

**Results:**

DEX significantly induced MKP-1 expression in both patients with SS and SR patients in a concentration-dependent manner, but greater induction was observed for SS patients at a low concentration (10^−6^ M). Asthmatic serum alone showed no MKP-1expression, and there was impaired induction of MKP-1 by DEX in SR asthma patients. The expression of GLCCI1 was not induced in neutrophils with DEX or DEX/atopic asthmatic serum combination. Greater inhibition of IL-8 production was observed in neutrophils from patients with SS asthma treated with DEX/atopic asthmatic serum combination compared with SR asthma patients, though DEX alone showed the same effect on neutrophils from SS and SR asthma patients. Meanwhile, DEX dependent inhibition of constitutive neutrophil apoptosis was similar between SS asthma and SR asthma patients.

**Conclusions:**

DEX exerted different effects on neutrophils from patients with SS asthma and SR asthma, which may contribute to glucocorticoid insensitivity.

## Background

Bronchial asthma is a chronic airway inflammatory disease that affects more than 300 million people worldwide, and this number is predicted to rise to 400 million by 2020 [1, 2]. The defining features of asthma are inflammation, airway hyperresponsiveness, reversible airway obstruction, and airway structural remodeling [3, 4]. A large array of cytokines, chemokines, and other pro-inflammatory mediators released by both immune-inflammatory and airway structural cells contribute to the pathophysiology asthma [5–7]. Considerable evidence shows that airway inflammation is a major factor in asthma pathogenesis, and that bronchial hyperresponsiveness in asthma often correlates with disease severity [8].

Glucocorticoids are potent anti-inflammatory drugs used as a mainstay treatment for asthma [9]. The anti-inflammatory effects of corticosteroids are mediated through the glucocorticoid receptor (GR), which binds to glucocorticoid response elements (GREs) to regulate the transcription of specific genes [10]. In most patients, asthma symptoms can be well controlled with low doses of inhaled corticosteroids (ICSs). However, some patients require higher doses of ICSs or even oral corticosteroids to achieve optimal control, indicating that these asthma patients might be relatively resistant to the anti-inflammatory actions of corticosteroids (steroid resistant asthma). Between 10 and 25% of asthmatics are steroid resistant (SR), and do not respond to glucocorticoids (GCs) therapy [11–13]. Several studies found that steroid resistant (SR) asthmatics do not have eosinophils in their airways, but instead have neutrophils [14].

The role of neutrophils in bronchial asthma pathogenesis is controversial [15]. Previous studies demonstrated that neutrophils play a regulatory role in asthma, since they synthesize and release an array of inflammatory mediators that trigger the development of asthma [16]. Severe asthma, persistent asthmatic status, acute asthma exacerbation, and corticosteroid-resistant asthma are associated with increased numbers of neutrophils in peripheral blood and bronchoalveolar lavage fluid (BALF), sputum or bronchial biopsy samples from asthma patients [17, 18]. Neutrophils are generally considered to be less responsive to GCs, since events involved in neutrophil activation, including adherence, chemotaxis, degranulation, and arachidonic acid metabolite release are not effectively inhibited by glucocorticoids [19]. Many studies showed that SR asthma was associated with impaired in vitro and in vivo responsiveness of monocytes and T lymphocytes to the suppressive effects of GCs [20–22], but little is known about the responsiveness of neutrophils. In this study, we examined whether neutrophils from SS asthmatics and SR asthmatics respond differently to corticosteroids in vitro.

## Methods

### Subjects

Thirty-eight adult asthmatic patients between the ages of 18 and 65 years were recruited from Tongji Hospital. The diagnosis of asthma was based on the Global Initiative for Asthma (GINA) guidelines. All subjects had a doctor’s diagnosis of symptomatic asthma and demonstrated evidence of airways hyperresponsiveness (PD20 methacholine < 2.5 mg) and/or bronchodilator responsiveness (>12% improvement in FEV1% predicted following inhalation of 200 μg salbutamol). Participants were excluded if they were current smokers or ex-smokers with a history more than 10 pack-years, had a course of oral corticosteroids, or a respiratory tract infection in the previous 4 weeks. The clinical characteristics of these participants are described in detail in Table 1. Patients’ clinical responses to corticosteroids were determined based on change in prebronchodilator FEV1 percent predicted after a one-month ICS treatment. Asthmatics were defined as SR if they had less than 15% improvement in baseline prebronchodilator FEV1. Among the 38 patients, 26 and 12 were classified as having SS and SR asthma, respectively. Blood samples were collected from all patients at their first visit of the study. Written informed consent was obtained from all subjects and the study was approved by the ethics committee of Tongji Hospital, Tongji Medical College, Huazhong University of Science and Technology.

**Table 1:** Patient characteristics

	Patients with SS asthmatic (*n* = 26)	Patients with SR asthmatic (*n* = 12)
Age(y), mean ± SD	43.81 ± 2.54	40.25 ± 2.9
Sex (male/female)	12/14	7/5
Duration of asthma (y), mean ± SD	4.92 ± 1.51	4.0 ± 1.83
Baseline FEV1 (% predicted), mean ± SD	70.77 ± 3.71	75.43 ± 4.18
FEV1/FVC (%), mean ± SD	61.62 ± 1.53	67.6 ± 3.97
FEV1 (% change after one month treatment), mean ± SD	35.65 ± 3.70	9.23 ± 1.15***

*FEV1* forced expiratory volume in 1 s, *FVC* forced vital capacity, *ICS* inhaled corticosteroid, *LABA* long-acting b-agonist

***P < 0.001 compared with patients with SS asthma

### Neutrophil isolation and cell culture

Neutrophils were separated from the heparinized peripheral blood of asthmatics using Ficoll-Hypaque gradient centrifugation. Briefly, peripheral blood was mixed with hydroxyethyl starch 550 (HES-TBD 550, TBDscience, Tianjin, China) and PBS, and allowed to sediment for 30 min. The leukocyte containing supernatant was then carefully layered onto Ficoll-Hypaque gradient. After centrifugation at 800 g for 25 min, all the layers above the red cells were removed. The cells were washed after hypotonic lysis to remove erythrocytes and then resuspended at 5 × 10^5^/ml in RPMI 1640 medium (GIBCO Laboratories, Grand Island, NY) with 1% penicillin-streptomycin (KeyGEN, Nanjing, China) and 10% FBS (GIBCO Laboratories, Grand Island, NY) or 10% atopic asthmatic serum. This method routinely yielded a purity >98% as determined by Wright-Giemsa staining. The atopic asthmatic serum was obtained from allergic asthmatic patients with serum IgE levels >1000 IU/ml. Isolated neutrophils were treated with dexamethasone (Sigma–Aldrich, St. Louis, MO USA) at a concentration of 10^−6 ^M or 10^−4^ M or left unstimulated for 18 h.

### Assessment of apoptosis

To detect neutrophil apoptosis, an annexinV–fluorescein isothiocyanate (FITC) apoptosis detection kit (KGA108, KeyGEN, Nanjing, China) was used according to the manufacturer’s protocol. Briefly, neutrophils were washed twice in ice-cold PBS and resuspended in 500 μl binding buffer containing 5 μl FITC-labeled annexin V and 5 μl propidium iodide (PI) for 15 min at room temperature in the dark. Apoptotic neutrophils were analyzed using a FACSCalibur with CellQuest software (BD Biosciences), and were determined as the percentage of cells showing annexin V+/PI- and annexin V+/PI+ staining.

### Enzyme-linked immunosorbent assay (ELISA)

The concentrations of IL-8 in cell culture supernatants were measured with a sandwich enzyme-linked immunosorbent assay (ELISA) using a Human IL-8 DuoSet ELISA kit (R&D Systems, Minneapolis, MN, USA) according to the manufacturer’s instructions. Briefly, a 96-well microplate was coated with capture antibody and incubated overnight at room temperature. Then, block plates by incubating with Block Buffer at room temperature for 1 h. The standards or cell supernatants were added to the plate; Detection Antibody, and streptavidin-HRP was added. After removal the unbound material by a washing procedure, substrate solution [1:1 mixture of color reagent A (H2O2) and color reagent B (tetramethylbenzidine)] was added in the dark. To stop the reaction, 2 N H2SO4 was added to each well. The optical density was determined at 450 nm and a reference wavelength of 570 nm using a spectrophotometer. Standard curve was constructed by plotting absorbance values versus the corresponding concentration of standard. Concentrations were calculated based on the standard curve. The limit of detection was 31.3 pg/mL.

### RNA isolation, reverse transcription and quantitative real-time PCR

Total RNA from cells was extracted using TRIzol reagent (Takara, Dalian, China) according to the manufacturer’s instructions using three-step nucleic acid precipitation with 0.2 volume of chloroform, 1 volume of isopropanol and 75% ethano. Extracted RNA was dissolved in 20 μL diethylpyrocarbonate-treated water. Total RNA concentration and purity were evaluated by spectrophotometry (NanoDrop 2000, Thermo scientific Fisher, Waltham, MA, USA). The cDNA was prepared from 500 ng total RNA using Prime Script RT Master Mix (Takara, Dalian, China) following the manufacturer’s instructions. Briefly, the reaction mixture (10 μL) containing 2 μL 5 × Prime Script RT Master Mix was cycled at 37 °C for 15 min, 85 °C for 5 s, and 4 °C for 1 min. Real-time PCR was performed to quantify mRNA levels using an ABI Prism 7500 Real-Time System (Applied Biosystems, Foster City, California, USA) with SYBR Premix Ex Taq (Takara, Dalian, China). 1 μL of cDNA was used in 20-μL final PCR volume containing 10 μL of SYBR Premix Ex Taq, 0.4 μL of ROX Reference Dye II, and 0.2 μM each sense and antisense primers. The PCR parameters were 95 °C for 30 s, followed by 40 cycles of 95 °C for 5 s and 60 °C for 34 s. Results were expressed as 2^–ΔΔCT^, normalized to levels of β-actin in untreated cells. Gene expression was reported as the relative variation (fold change) to unstimulated cell mRNA levels. The primers used in this study were synthesized by Sangon Biotech (Shanghai, China) and had the following sequences: β-actin (forward, GCAAGCAGGACTATGACGAG and reverse, CAAATAAAGCCATGCCAATC); GLCCI1 (forward, GGGAAGGAAGAAGTATCCAAGC and reverse, GCGAGTACTACTGCTCCGGTA); MKP1 (forward, GCTGTGCAGCAAACAGTCGA and reverse, CGATTAGTCCTCATAAGGTA).

### Statistical analysis

Data were expressed as means ± SD. All statistical analyses were conducted using GraphPad Prism 5 software (GraphPad, San Diego, CA, USA). Data were analyzed by t test if the data followed a normal distribution; nonparametric tests were applied for the data that were not normally distributed. All statistical tests were two-tailed and differences were considered significant at a *P* value of less than 0.05.

## Results

### Study subjects

Clinical characteristics of the study participants are summarized in Table 1. In this study patients were defined as having SR or SS asthma based on changes in lung function after 1 month of ICS treatment. Asthmatics were defined as SR if they had less than 15% improvement in baseline prebronchodilator FEV1. The patients with SS and SR asthma were similar in terms of demographics, duration of asthma, and mean FEV1 at baseline. The only clinical difference between the two patient group was changes in lung function after ICS (mean △FEV1 percent predicted); the patients with SS asthma showed significant improvement in △FEV1 (Mean ± SD) of 35.65 ± 3.70%, whereas no significant change in FEV1% predicted was noted in the patients with SR asthma with a △FEV1 (Mean ± SD) of 9.23 ± 1.15%.

### In vitro markers of corticosteroid responsiveness

**Fig. 1:**
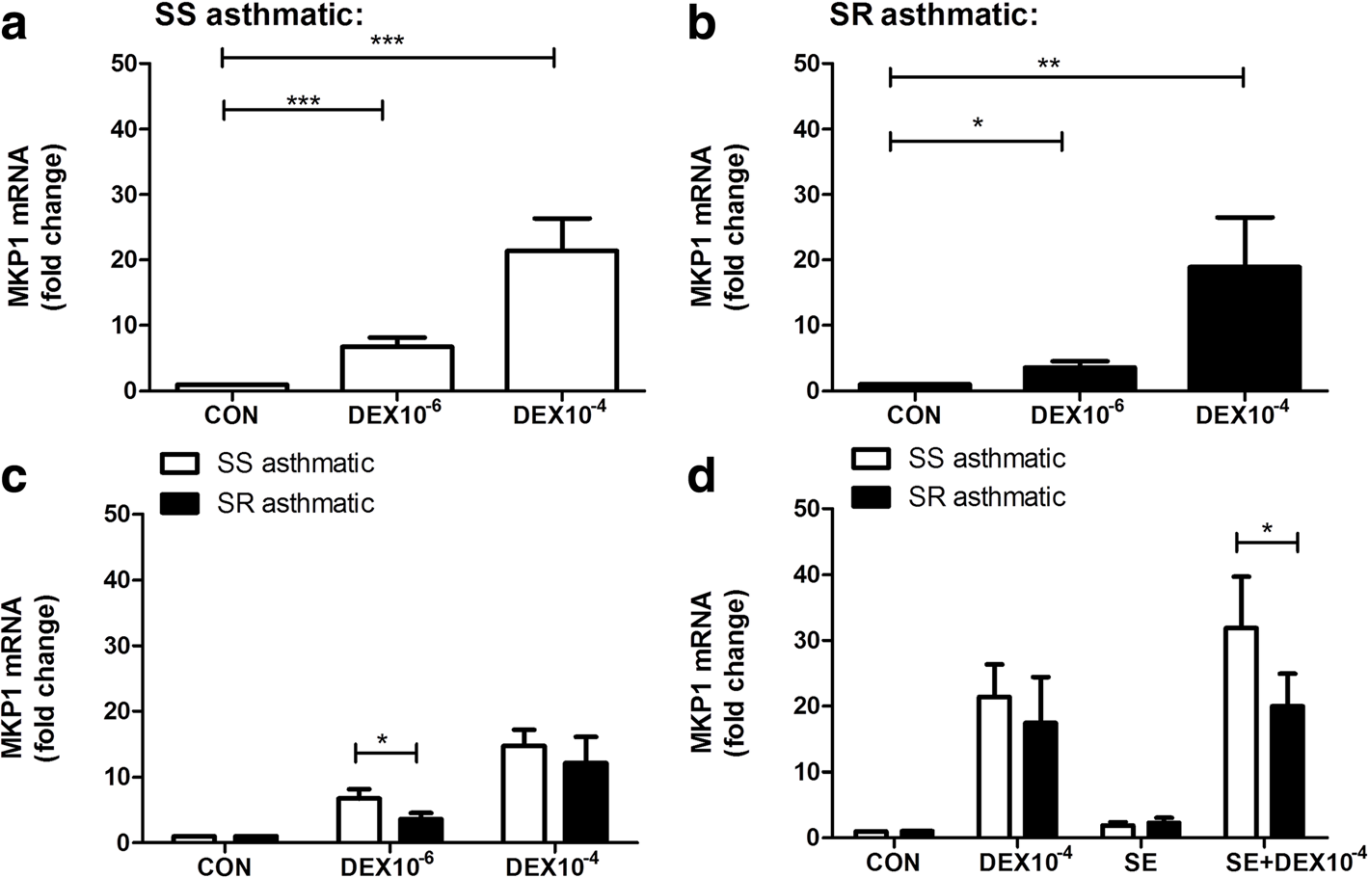
MKP1 gene expression induced by dexamethasone (DEX) in asthmatic patients. Neutrophils were isolated from peripheral blood of asthmatic patients and then incubated for 18 h in the absence (Con) or presence of dexamethasone (DEX) at 10^−6^ M or 10^−4^ M, with/without atopic asthma serum (SE). Following culture, mRNA expression of MKP1 was quantified by realtime-PCR. **a** and **b**, Neutr﻿ophils respectively from SS asthmatics (**a**) and SR asthmatics (**b**) were ﻿inc﻿ubated with dexamethasone (DEX) at 10^−6^ M or 10^−4^ M. **c**, MKP-1 induction by DEX at 10^−6^ M was significa﻿ntly gre﻿ater from SS a﻿sthmatics. **d**, Neu﻿trophils were isolated from a﻿sthmatic pat﻿i﻿ents and incubated with DE﻿X 10^−4 ^M, and/o﻿r asthmatic serum (SE). * *P* < 0.05, ** *P* < 0.01, and *** *P* < 0.001 compared with the control groups

Neutrophils were isolated from patients’ blood at their first visit. The expression of selected gene targets was analyzed by real time PCR after treating cells with 10^−6^ M or 10^−4^ M DEX, or DEX/asthmatic serum for 18 h. MKP-1 was selected as a well-known glucocorticoid transactivation target [23]. DEX significantly induced MKP-1 expression in neutrophils from SS asthma patients in a concentration-dependent manner to produce a 6.18 ± 1.46 (*P* < 0.0001) and 21.38 ± 4.96-fold (*P* < 0.0001) at 10^−6^ M and at 10^−4^ M DEX, respectively,relative to control cells (Fig. 1a). Similarly, DEX significantly increased MKP-1 levels in neutrophils from patients with SR asthma by 3.61 ± 0.94-fold (*P* < 0.05) at 10^−6^ M and 18.88 ± 7.62-fold (*P* < 0.01) at 10^−4^ M relative to control cells (Fig. 1b). Notably, the level of MKP-1 induced by 10^−6^ M DEX was significantly greater in neutrophils from patients with SS asthma relative to patients with SR asthma (*P* < 0.05) (Fig. 1c). Asthmatic serum alone showed no MKP-1 expression, but when combined with DEX, asthmatic serum impaired the induction of MKP-1 by DEX in neutrophils from SR asthma patients (*P* < 0.05). (Fig. 1d).

### The effects of DEX on GLCCI 1 gene expression in SS and SR asthmatic patients

**Fig. 2:**
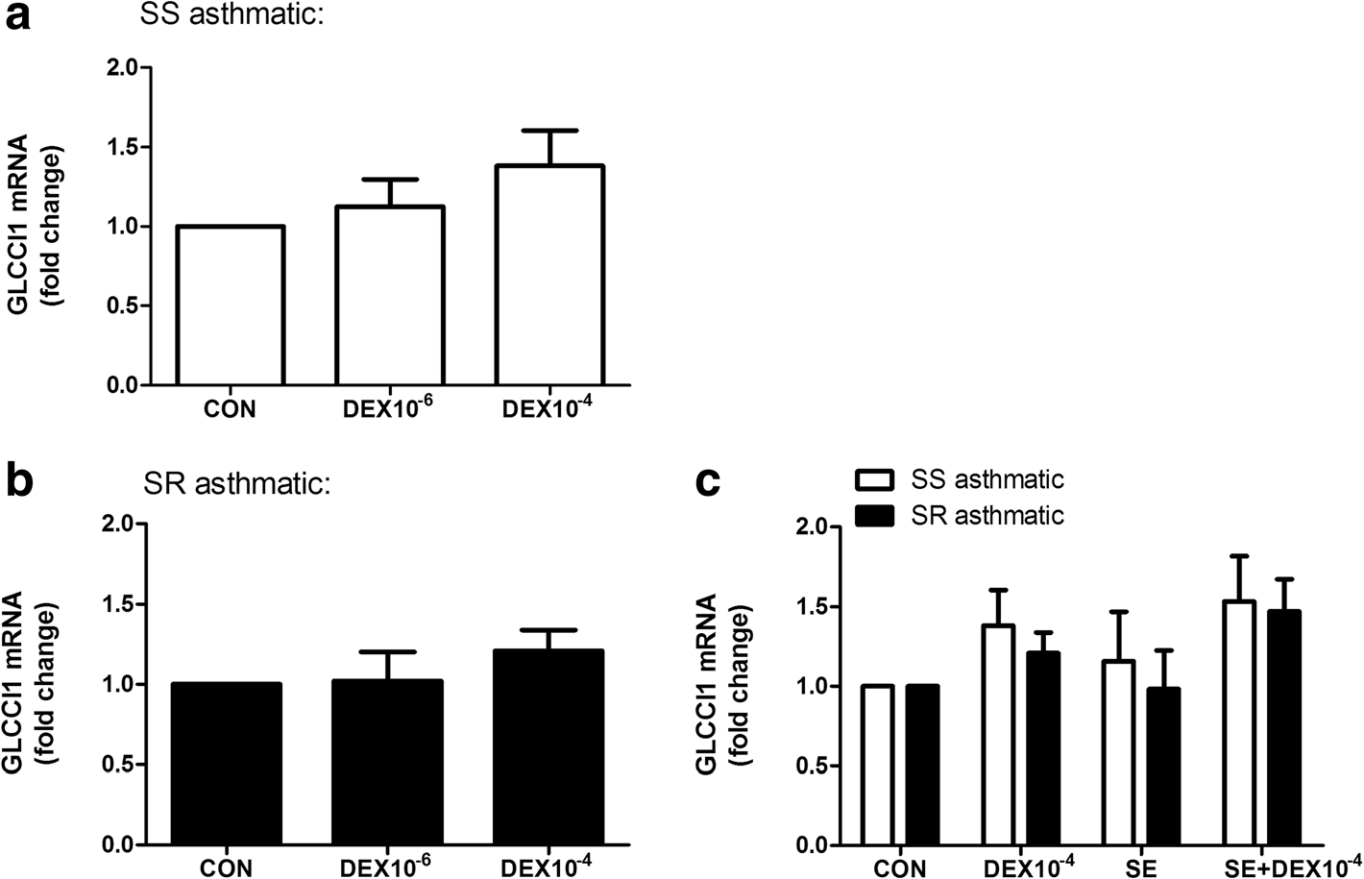
The effects of dexamethasone on GLCCI1 gene expression by neutrophils from asthmatic patients. Neutrophils were isolated from peripheral blood of asthmatic patients and then incubated for 18 h in the absence (Con) and presence of asthmatic serum and/or dexamethasone (DEX) at 10^−6^ M or 10^−4^ M. Following culture, GLCCI1 mRNA expression was quantified by realtime-PCR. **a** and **b**, Neutr﻿ophils respectively from SS asthmatics (**a**) and SR asthmatics (**b**) were ﻿inc﻿ubated with dexamethasone (DEX) at 10^−6^ M or 10^−4^ M. **c**, N﻿eutr﻿ophi﻿ls were isolated from asthmatic patients and incubated with DEX 10^−4 ^M, a﻿nd/o﻿r asthmatic serum (SE). * *P* < 0.05 compared with the control groups

Glucocorticoid-induced transcript 1 (GLCCI1) is expressed in both lung cells and immune cells, and its expression is significantly enhanced by the presence of glucocorticoids in asthma-like conditions [24]. Here we examined the expression of GLCCI1 mRNA in response to DEX with or without asthmatic serum in neutrophils isolated from SS and SR asthma patients. Neutrophils from participants treated with 10^−6^ M or 10^−4^ M DEX without asthmatic serum or DEX/asthmatic serum for 18 h. There was no significant change in the level of GLCCI1 in neutrophils treated with DEX from SS asthma and SR asthma patients (Fig. 2a and b). Similarly, the levels of GLCCI1 were not affected by DEX/asthmatic serum combination in SS asthma and SR asthma patients (Fig. 2c).

### DEX inhibited IL-8 production by neutrophils from SS asthmatics and SR asthmatics

**Fig. 3:**
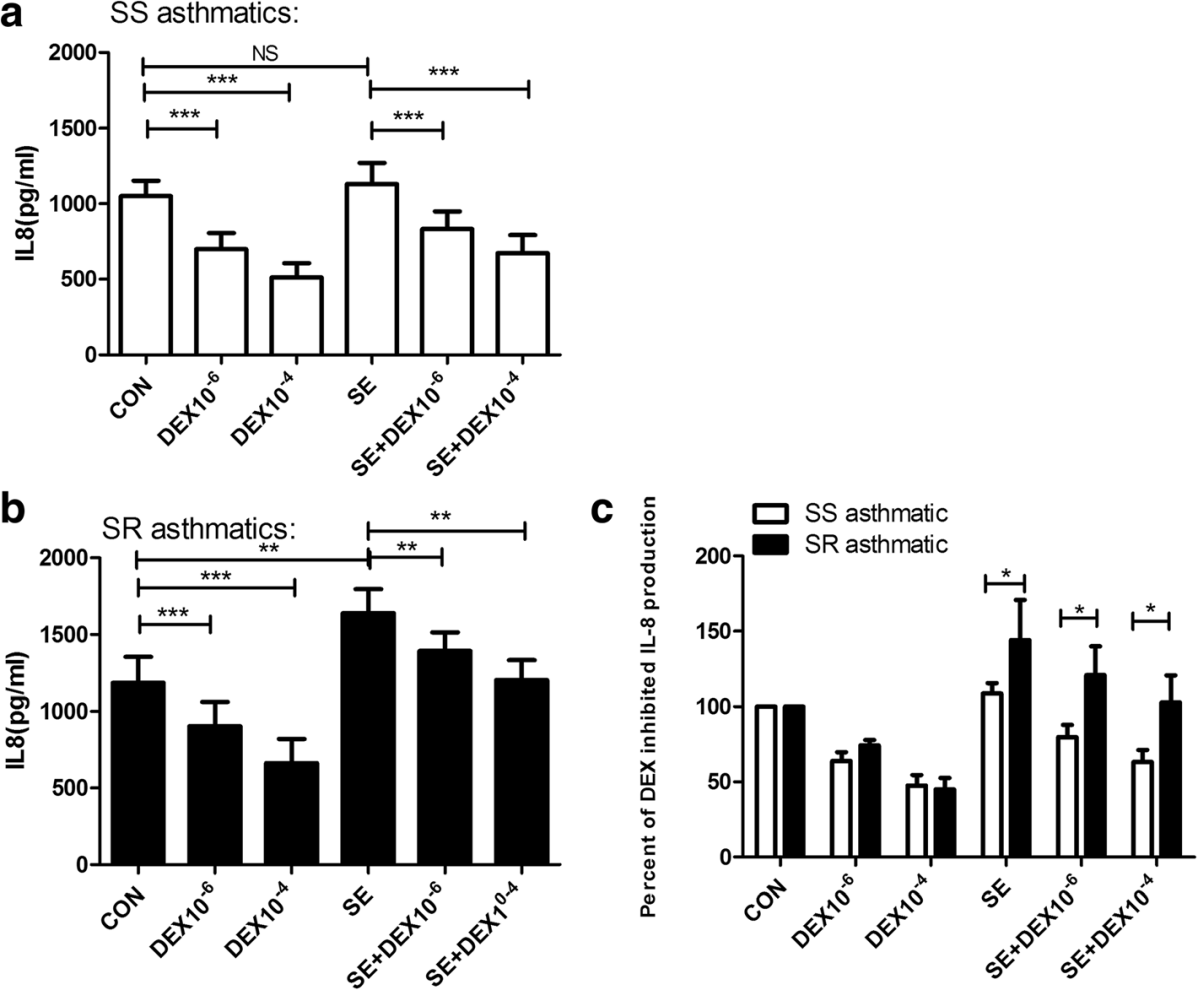
Cytokine release was inhibited by dexamethasone in patients with SS asthma and SR asthma. Neutrophils from peripheral blood of asthmatic patients(**a**, SS asthmatics; **b**, S﻿﻿R asthmatics) were incubated with dexamethasone (DEX) at 10^−6^ M or 10^−4^ M and/or asthmatic serum (SE) for 18 h. The supernatant was collected and analyzed by ELISA. Data are expressed as the means ± SD (**a**, **b**). **c**, Percentages of IL-8 levels for DEX or asthmatic serum compared with the amount of IL-8 produced by untreated cells (100%) are shown. * *P* < 0.05, ** *P* < 0.01, and *** *P* < 0.001 compared with the control groups

The capacity of neutrophils to produce IL-8 has been documented both in vitro and in vivo and is a useful indicator of protein synthesis by activated cells [25]. We measured IL-8 production in culture to examine if DEX had different effects on activation of neutrophils isolated from SS and SR asthma patients. Neutrophils from SS asthmatics and SR asthmatics were incubated with 10^−6^ M or 10^−4^ M DEX either with or without asthmatic serum for 18 h. IL-8 levels in cell culture supernatants were then assessed by ELISA. Untreated cells produced detectable IL-8 levels, and this level increased in the presence of asthmatics serum from a basal level of 1183 ± 171.5 pg/mL to 1640 ± 156.3 pg/mL for SR asthma patients (Fig. 3). However, there was no significant change in SS asthma patients. Meanwhile, DEX significantly and dose-dependently reduced IL-8 production in neutrophils from patients with SS asthma and SR asthma. The inhibition was similar between SS asthma and SR asthma with either DEX at 10^−6^ M or 10^−4^ M (Fig. 3c). However, the asthmatics serum impaired the inhibitory activity of DEX towards neutrophils from SR asthma (Fig. 3c). Significantly greater inhibition of IL-8 production was observed with neutrophils from patients with SS asthma in the presence of DEX/asthmatic serum combination when compared with neutrophils of patients with SR asthma (*P* < 0 .05).

**Fig. 4:**
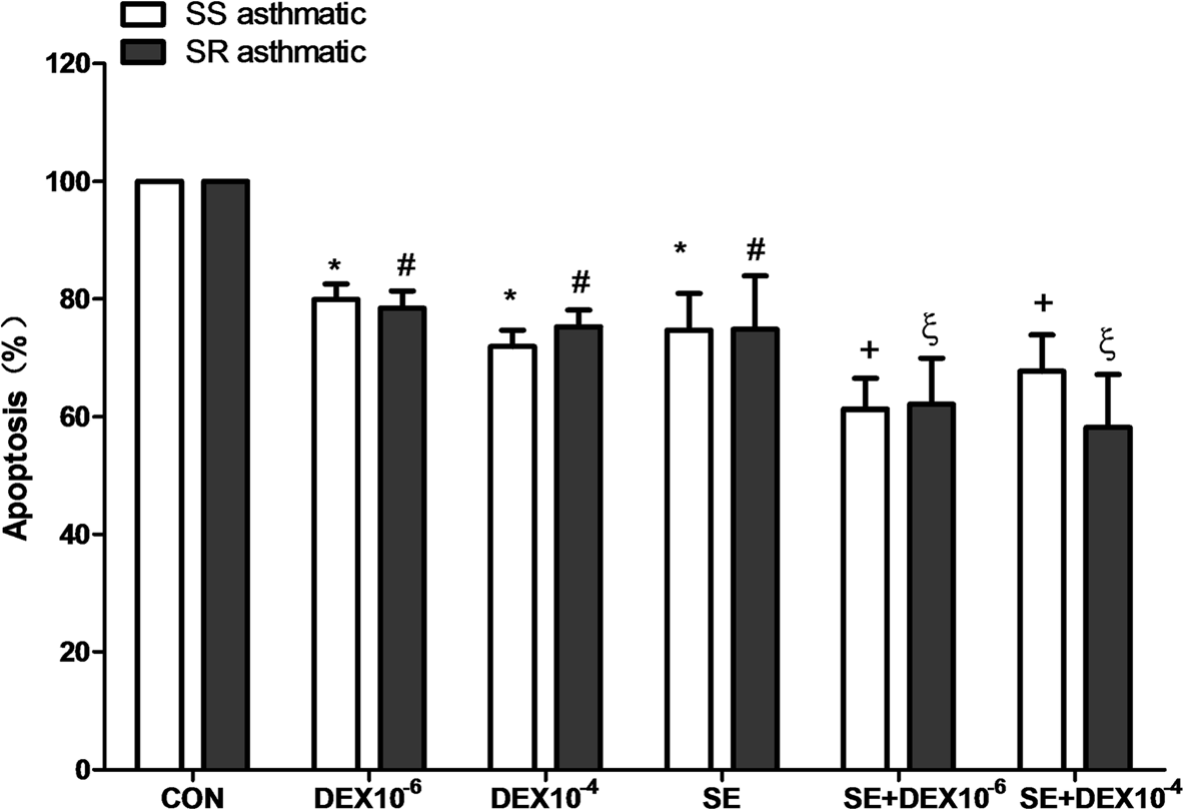
Comparison apoptosis of neutrophils from SS asthma and SR asthma patients. Neutrophils were isolated from the peripheral blood of asthmatic patients and then incubated for 18 h in the absence (Con) or presence of asthmatic serum and/or dexamethasone (DEX) at 10^−6^ M or 10^−4^ M. Apoptosis was analyzed by measuring the binding of annexin V-FITC and PI. Data are presented in relation to the control, which was set at 100%. Data was expressed as the means ± SD. * *P* < 0.05 compared with the SS asthmatic control groups, # *P* < 0.05 compared with the SR asthmatic control groups, + *P* < 0.05 compared with the SS asthmatic treated with asthmatic serum groups (SE), ξ *P* < 0.05 compared with the SR asthmatic treated with asthmatic serum groups (SE)

### DEX inhibited constitutive apoptosis of neutrophils from SS and SR asthma patients despite the presence of asthmatic serum

We next evaluated whether the effect of DEX on constitutive apoptosis of neutrophils in the presence or absence of asthmatic serum differs between SS and SR asthma patients. Constitutive apoptosis of neutrophils from SS asthma patients was inhibited by both 10^−6^ M and 10^−4^ M DEX, as was apoptosis of neutrophils from SR asthma patients (Fig. 4). Meanwhile, the addition of asthmatic serum had no effect on DEX inhibition of apoptosis of neutrophils from SS and SR asthma patients (Fig. 4).

## Discussion

Here we examined the effect of DEX on neutrophils from SS asthmatics and SR asthmatics. The expression of selected markers in neutrophils that reflect the response to DEX significantly differed between SR and SS asthmatics. The ability of DEX to induce MKP-1 expression was significantly reduced in neutrophils from SR asthmatics compared to those from SS asthmatics. Additionally, the level of GLCCI1, which is significantly associated with the response of asthma patients to inhaled glucocorticoids [24], was not affected by DEX in neutrophils from asthmatics. We also assessed IL-8 production of neutrophil, an indicator of neutrophil activation [25]. The data demonstrated that more DEX was required to suppress IL-8 production by SR asthmatic neutrophils relative to SS asthmatics when asthmatic serum was added. We next demonstrated that DEX exerted a similar effect on constitutive apoptosis of neutrophils from SR and SS asthmatics. Thus, we concluded that neutrophils from SR asthma patients were less responsive to DEX, but the constitutive apoptosis was comparable to that for neutrophils from SS asthmatics.

SR asthmatics are characterized by persistent airway inflammation despite treatment with corticosteroids, and therefore these patients could be predisposed to increased airway remodeling and irreversible lung disease [11, 26, 27]. Given the increasing prevalence and severity of asthma worldwide, steroid resistance has become a challenging health problem that imposes enormous burdens on health care [28, 29]. Therefore knowledge of the mechanisms by which corticosteroids have differential effects in SS and SR asthma patients is indispensable.

MKP-1 is a phosphatase that dephosphorylates and inactivates MAPKs, including p38 mitogen-activated protein kinase (MAPK) [23], and inhibits production of pro-inflammatory cytokines, which is critical for the anti-inflammatory functions of corticosteroids [30]. Recent studies demonstrated increased activation of p38 MAPK in peripheral blood monocytes from SR asthmatics compared to those from SS asthmatics [31]. MKP-1 has also been identified by as a marker of in vitro responsiveness to corticosteroid treatment [20]. These observations prompted us to determine changes in MKP-1 expression levels in DEX-treated neutrophils isolated from asthmatic patients. DEX significantly induced MKP-1 expression in neutrophils from asthmatics, and this induction is similar to that seen for other cell types, including mast cells [32], macrophages [33], osteoblasts [34], monocytes [22] and airway smooth muscle cells (ASMC) [35]. The induction of MKP-1 expression by DEX at 10^−6^ M in neutrophils from SS asthma patients was higher than that for SR asthma patients. The sensitivity to glucocorticoids is correlated with MKP-1 inducibility, which provides support for the use of MKP-1 as a potential determinant of corticosteroid responsiveness. This possibility is consistent with a previous observation that of increased activation of p38 MAPK in airway macrophages isolated from severe asthmatics compared to those from non-severe asthmatics, and that the severe asthmatic group had impaired corticosteroid induction of MKP-1 [36]. Atopic asthmatic serum has been used to mimic the asthmatic milieu to sensitize airway smooth muscle cells (ASMC) [37, 38]. In this study, we used atopic asthmatic serum to passively sensitize neutrophils to simulate asthma conditions in vitro. Atopic asthmatic serum alone had no effect on MKP-1 expression by neutrophils, while neutrophil with both atopic asthmatic serum and DEX impaired DEX-induced MKP-1 expression in SR asthma patients. These data suggest that the ability of asthma milieu to reduce DEX-induced MKP-1 transcription in neutrophils from SR asthma patients may be associated with corticosteroid insensitivity.

Insensitivity to glucocorticoid is an important issue in asthma management, and may lead to poor asthma control and deterioration of airflow. Whether molecular glucocorticoid responses have a genetic component has been extensively examined [39–41]. GLCCI1 was identified as a novel pharmacogenetic determinant of the response of asthma patients to inhaled corticosteroid [24]. Indeed, Tantisira et al. reported that a functional polymorphism of GLCCI1 is associated with a substantially decreased response to inhaled glucocorticoids in patients with asthma [24]. GLCCI1 expression is rapidly induced by glucocorticoids in murine thymocytes [42]. However, here the GLCCI1 inducibility by DEX in neutrophils from asthma patients was invalid . There was no statistically significant significance in the level of GLCCI1 in SS and SR asthma neutrophils treated with DEX or asthmatic serum/DEX. This finding is consistent with previous data, wherein DEX inhibited apoptosis of neutrophils from patients with SS asthma and SR asthma to the same extent. GLCCI1 is thought to be an early marker of glucocorticoid-induced apoptosis [42]. The neutrophil apoptosis suppressed by DEX may contribute to the apparent lack of GLCCI1 expression induction.

Neutrophils are generally considered to be less sensitive to GC. However, previous findings confirmed that GCs inhibit pro-inflammatory gene expression in neutrophils from different species [43–45]. IL-8 is a powerful chemoattractant and activator of neutrophils that is released by a variety of cells, including neutrophils [16], and can therefore contribute to additional recruitment and activation of neutrophils in a positive feedback manner if its expression is not tightly regulated. The gene or protein expression of IL-8 is increased in asthma patients who are insensitive to glucocorticoid treatment, as is the case for severe asthmatic patients [46, 47] and patients with neutrophilic asthma [48, 49]. We thus investigated whether differential suppression of MKP-1 expression in SS and SR asthma patients by DEX influenced IL-8 release from neutrophils. The results showed a similar inhibition of IL-8 by DEX in SS asthma and SR asthma patients, but a significantly higher inhibition was observed in neutrophils from SS than SR asthma patients treated with the DEX/asthmatic serum combination, which is consistent with the MKP-1 induction data. The insensitivity to GC of SR asthma neutrophils in the presence of a mimetic asthma milieu should also contribute to lowered responsiveness to the inhibition of pro-inflammatory cytokine release promoted by DEX.

Inducing inflammatory cell apoptosis is a key mechanism through which glucocorticoids resolve lymphocytic and eosinophilic inflammation in asthma [50]. Corticosteroids reduce the rate of neutrophil apoptosis to prolong their survival [25, 51]. In our study, DEX inhibited constitutive neutrophil apoptosis in SS asthma and SR asthma patients to the same extent. Atopic asthmatic serum slightly reduced neutrophil apoptosis in the two groups, but there was no significant difference between SS asthma and SR asthma in the presence of atopic asthmatic serum alone or in combination with DEX. Thus, the apoptosis that is inhibited by DEX is not associated with insensitivity to glucocorticoid. The increased number of neutrophils in the airways of patients with severe asthma [52], may contribute to corticosteroid resistance or refractory disease, and could be due to the lessened inhibition of pro-inflammatory cytokine release rather than apoptosis inhibited by DEX.

## Conclusions

DEX exerted different effects on neutrophils from SS asthma and SR asthma patients. No significant differences were seen in the apoptosis of neutrophils between patients with SS asthma and SR asthma, and therefore it is unlikely that glucocorticoid sensitivity is associated with the inhibition of apoptosis by glucocorticoid. DEX induced MKP-1expression and inhibited IL-8 production by neutrophils, but a reduced MKP-1 mRNA induction and IL-8 inhibition in response to DEX/asthmatic serum combination was observed in patients with SR asthma. This dysfunction may contribute to the insensitivity to glucocorticoid of these patients.

## Abbreviations

ASMC: Airway smooth muscle cells
BALF: Bronchoalveolar lavage fluid
DEX: Dexamethasone
ELISA: Enzyme-linked immunosorbent assay
FEV1: Forced expiratory volume in one second
FITC: Fluorescein isothiocyanate
FVC: Forced vital capacity
GC: Glucocorticoid
GINA: Global Initiative for Asthma
GR: Glucocorticoid receptor
GRE: Glucocorticoid response element
ICS: Inhaled corticosteroid
LABA: Long-acting b-agonist
GLCCI1: Glucocorticoid-induced transcript 1
MAPK: Mitogen-activated protein kinase
MKP-1: Mitogen-activated protein kinase phosphatase-1
PD20: Dose producing a decrease of 20% from base line in FEV1
PI: Propidium iodide
SD: Standard deviation
SR: Steroid resistant
SS: Steroid sensitive
